# Supplementary material for: Na+/Ca2+ exchangers and Orai channels jointly refill endoplasmic reticulum (ER) Ca2+ via ER nanojunctions in vascular endothelial cells
Source: Pflugers Arch. 2017 May 11;469(10):1287–99. doi: 10.1007/s00424-017-1989-8 (PMC5590033; doi:10.1007/s00424-017-1989-8)
Supplement: Supplementary file 1 — (PDF 610 KB) [file 424_2017_1989_MOESM1_ESM.pdf]

Supplementary Material

Cristiana M. L. Di Giuro, Niroj Shrestha, Roland Malli, Klaus Groschner,

Cornelis van Breemen, and Nicola Fameli

S1 Control experiment for influence of transfection on recorded Ca^{2+} signals

In a separate set of experiments, we subjected a population of EA.hy926 cells to sham transfection in order to verify whether or not the Ca^{2+} signal would be affected by the transfection process itself. After analyzing the data as described in the Materials and Methods section of the main article, we found virtually no difference between the sham-transfected and the non-transfected sets of recorded traces, as can be seen in figure S1.

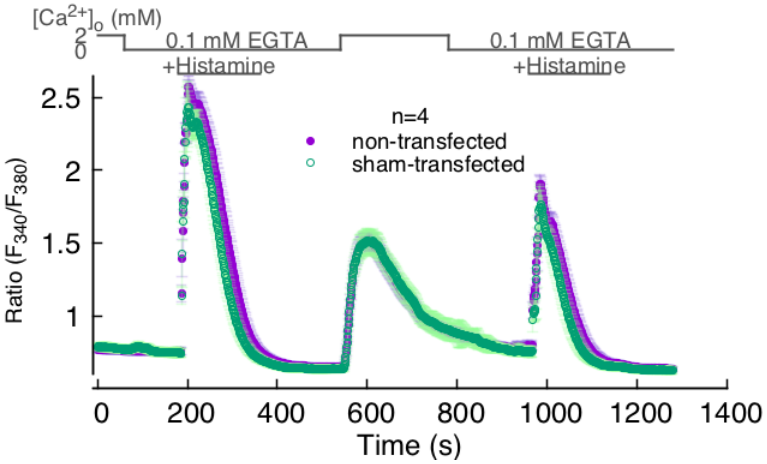

Figure S1: Fura-2 signal traces from non-transfected (solid purple circles) and sham-transfected (empty green circles) cells.

S2 ER Ca^{2+} releasable fraction in absence of normal Ca^{2+} phase (nCa^{2+})

The data reported in the solid red circles and red bar in figure S2A and B are the same as in figure 2C and D (also in red) in the main article. They represent the Fura-2 signal during a series of experiments in absence of the nCa^{2+} phase of the protocol (see top part of figure S2A).

In the experiments performed to obtain the data in empty green circles in figure S2A and B, we added the reversible SERCA blocker 2,5-Di-tert-butyl-1,4-benzoquinone (BHQ) during the first histamine stimulation period to prevent the Ca^{2+} released from the ER from being re-uptaken while still present in free form in the cytosol.

These results show that the majority of the ER releasable Ca^{2+} remaining after a 3-min histamine stimulation is due to internal Ca^{2+} "recycling" during the stimulation itself and confirm that the ER releasable Ca^{2+} fraction is virtually entirely refilled from the extra-cellular space.

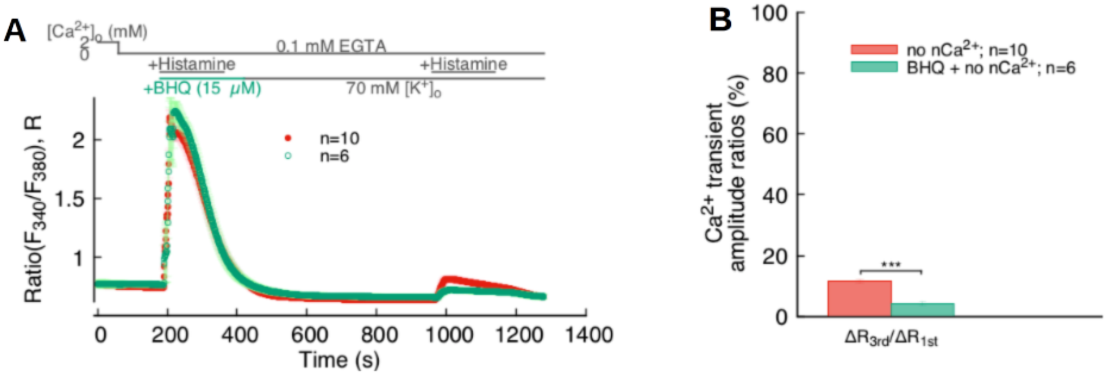

Figure S2: **A.** Fura-2 signal traces showing the effect of nCa^{2+} phase omission on the Ca^{2+} signal (solid red circles) and of the addition of 15μM BHQ during the first histamine stimulation in the same protocol (empty green circles); **B.** Bar chart from traces in A. *** indicates p<0.001.

S3 Smearing of the PM in the electron micrographs of PM-ER nanojunctions

The identification and characterization of PM-ER junctions in cultured cells like the EA.hy926 endothelial cells employed in our experiments can be hampered by the smearing of the PM due to the microtome sectioning phase of the sample preparation.

In figure S3 we report samples of micrographs from the set we collected showing instances of PM-ER nanojunctions, in which the PM is well defined (green arrow) thereby making measurement of the junctional gap and extension possible. In cases when the PM results smeared (red arrows) this is not possible and therefore the number of junctions we report in the histograms of the main article is likely an underestimate of the actual number.

The smearing effect is probably due to the fact that cultured cells lie flat on the sample substrate and present a cross-section that is bulbous at the location of the nucleus and gets progressively thinner toward the cell periphery. In these conditions, when sectioning the samples in a direction parallel to the cell substrate it becomes less likely that the knife cuts the PM as cleanly as other intracellular membranes lying in the thicker part of the cells. For this reason, we also opted to re-orient the samples so that they could be sectioned in a direction perpendicular to the substrate plane.

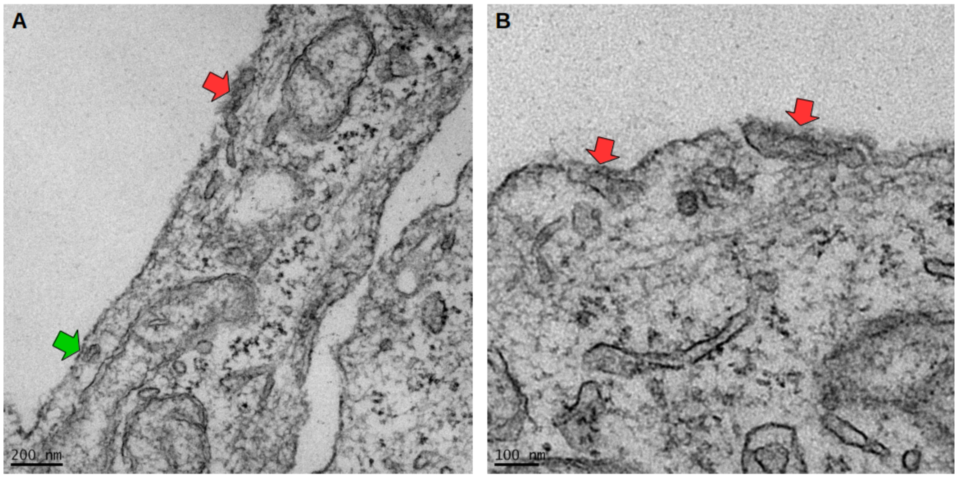

Figure S3: **A and B.** Representative electron micrographs of peripheral regions of EA.hy926 cultured endothelial cells showing evidence of PM-ER junctions with identifiable (green arrow) and smeared (red arrows) PM sections.

S4 TPRC channels appear not to contribute to ER Ca^{2+} refilling

Transient receptor potential canonical (TRPC) channels appear to contribute only to the bulk cytosolic Ca^{2+} in EA.hy926 endothelial cells, but not measurably to the ER Ca^{2+} content. In normally polarized cells (empty black circles in figure S4), Ca^{2+} entry via TRPC is virtually unimpaired (except perhaps for some interference by the unspecificity of KB-R7943). In this situation, while inhibiting NCX Ca^{2+} entry mode (with 10 μM KB-R7943), our data show that the Ca^{2+} amplitude transient ratio between the cytosolic signal and the ER Ca^{2+} releasable fraction is significantly higher than in depolarized cells ($\Delta R_{2nd}/\Delta R_{3rd}$ in figure S4), while the ER releasable Ca^{2+} fraction is not appreciably different ($\Delta R_{3rd}/\Delta R_{1st}$ in figure S4).

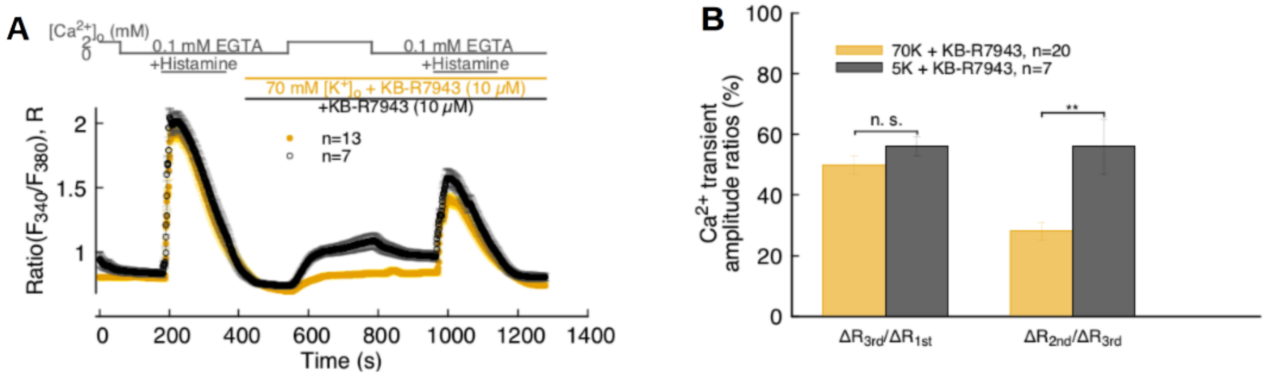

Figure S4: **A.** Fura-2 signal traces showing the effect of NCX inhibition by 10 μM KB-R7943 on normally polarized EA.hy926 endothelial cells (empty black circles) compared to its depolarized counterpart (solid yellow circles), as shown in figure 3 in the main article); **B.** Bar chart from traces in A. ** indicates $p<0.01$.
